# Protectin DX restores Treg/T_h_17 cell balance in rheumatoid arthritis by inhibiting NLRP3 inflammasome via miR-20a

**DOI:** 10.1038/s41419-021-03562-6

**Published:** 2021-03-15

**Authors:** Shengwei Jin, Siyuan Sun, Hanzhi Ling, Jinglan Ma, Xu Zhang, Zhen Xie, Ning Zhan, Wenjie Zheng, Man Li, Yang Qin, Heping Zhao, Yan Chen, Xinyu Yang, Jianguang Wang

**Affiliations:** 1 Department of Anesthesia and Critical Care, The Second Affiliated Hospital of Wenzhou Medical University, Wenzhou, China; 2 Department of Biochemistry, School of Basic Medical Sciences, Wenzhou Medical University, Wenzhou, China; 3 Department of Pediatric Nephrology and Rheumatology, The Second Affiliated Hospital of Wenzhou Medical University, Wenzhou, China; 4 Department of Medicinal Chemistry, School of Pharmaceutical Sciences, Wenzhou Medical University, Wenzhou, China

**Keywords:** Autoimmunity, Immunopathogenesis, Chronic inflammation, Inflammasome

## Abstract

Regulatory T-cell (Treg)/T-helper 17 (T_h_17) cell balance plays an important role in the progression of rheumatoid arthritis (RA). Our study explored the protective effect of protectin DX (PDX), which restored Treg/T_h_17 cell balance in RA, and the role of the nucleotide-binding domain (NOD)–like receptor protein 3 (NLRP3) inflammasome pathway in this process. Using mass spectrometry, we discovered that level of PDX decreased in active-RA patients and increased in inactive-RA patients compared with HCs, and serum PDX was a potential biomarker in RA activity detection (area under the curve [AUC] = 0.86). In addition, a collagen-induced arthritis (CIA) mice model was constructed and PDX obviously delayed RA progression in the CIA model, upregulating Tregs and anti-inflammatory cytokines while downregulating T_h_17 cells and pro-inflammatory cytokines. Moreover, NLRP3 knockout and rescue experiments demonstrated that NLRP3 participated in PDX-mediated Treg/T_h_17 cell balance restoration, joint injury amelioration and inflammatory-response attenuation using *Nlrp3*^−/−^ mice. Furthermore, microarray and verified experiments confirmed that PDX reduced NLRP3 expression via miRNA-20a (miR-20a). In summary, we confirmed for the first time that PDX could effectively ameliorate CIA progression by restoring Treg/T_h_17 cell balance, which was mediated by inhibition of the NLRP3 inflammasome pathway via miR-20a.

## Introduction

Rheumatoid arthritis (RA) is a chronic autoimmune disease that progressively destroys the articular cartilage and bones of the joints^1^. Although its precise pathogenesis remains unclear, studies indicate that regulatory T-cell (Treg)/T-helper 17 (T_h_17) cell imbalance might play a role in the progression of RA^2^. Notoriously, T_h_17 cells mediate the pro-inflammatory response via interleukin-17A (IL-17A) and tumor necrosis factor alpha (TNF-α) secretion, which leads to tissue destruction as well as damage to articular cartilage and bone;^3^ meanwhile, Tregs mediate the anti-inflammatory response via IL-10 and TGF-β secretion, which maintains a state of autoimmune tolerance^4,5^. The T_h_17 cell pathway has been linked to multiple autoimmune diseases, and therapeutic inhibitors of the pathway show variably beneficial effects in RA. Moreover, a decrease in Tregs in RA development has been demonstrated, and the functional stability of Tregs in rheumatoid joints has also been iteratively confirmed^6^. Additionally, it is reported that Treg/T_h_17 cell balance might be regulated via the signal transducer and activator of transcription (*STAT*)/nucleotide-binding domain (NOD)–like receptor protein 3 (NLRP3) axis or by nuclear factor κ-light-chain-enhancer of activated B cells (NF-κB) signaling in a collagen-induced arthritis (CIA) model^7,8^. Considering all of the above, further investigation into the mechanism of Treg/T_h_17 cell balance regulation seemed valuable to discovering the potential pathogenesis and therapeutic target of RA.

Protectin DX (PDX) is a protectin D1 isomer belonging to the specialized pro-resolving mediator (SPM) family and is derived from ω−3 long-chain polyunsaturated fatty acids (ω−3 LC-PUFAs)^9^. Our previous study indicated that two other SPMs, maresin 1 (MaR1) and resolvin D1 (RvD1), moderated the inflammatory response in RA^10,11^. As for protectin D1, Sano found that it was increased in synovial fluid in RA^12^. It is also reported that protectin D1 inhibits secretion of TNF-α, as a pro-inflammatory cytokine that enhances the progression of RA^13^. Although Bazan and Serhan detected a decrease in PDX in pneumonia and obesity, which accompanied chronic inflammatory responses such as IL-10 downregulation and IL-1β upregulation, PDX’s ability to resolve inflammation in RA remains unknown^14,15^. Meanwhile, our previous study indicated that MaR1 acted to restore Treg/T_h_17 cell imbalance^11^. PDX in particular is a classic regulator that inhibits circulating B cells, but its influence on T_h_17 cell differentiation is unclear^16^. All of the above facts indicated a profound need to explore the protective role of PDX in RA, as well as the exact mechanism thereof.

In a recent study, Yu et al. confirmed that NLRP3 activation promotes differentiation of T_h_17 cells in mice with lupus^17^. Meanwhile, Park et al. demonstrated that NLRP3 negatively regulates Treg differentiation through karyopherin subunit alpha 2 (KPNA2)–mediated nuclear translocation^18^. These facts suggested that NLRP3 might participate in the regulation of Treg/T_h_17 cell balance. NLRP3 inflammasome is a multi-protein complex composed of NLRP3, apoptosis-associated speck-like protein containing a caspase activation and recruitment domain (ASC) and Caspase-1 (CASP-1), regulated by the toll-like receptor (TLR4) or CASP-11 signaling pathway to direct the differentiation of immune cells^19^. Studies have demonstrated that the modulation of NLRP3 inflammasome in macrophages is a mechanism in SPM-regulated inflammation resolution^20^. RvD2 in particular can inhibit CASP-1 activity in lipopolysaccharide (LPS)/palmitate–elicited peritoneal macrophages^20^. Therefore, we naturally wondered whether PDX directed T-cell differentiation by inhibiting NLRP3 in a similar way, which needed further experimental exploration.

Micro–ribonucleic acids (miRNAs) are noncoding RNAs that regulate the translation of proteins by silencing messenger RNA (mRNA)^21^. Our previous studies confirmed that MaR1 upregulates miR-21 to modulate Treg/T_h_17 cell balance in RA patients^11^. RvD1 is also confirmed to upregulate miRNA (miR)–146a-5p to suppress the expression of inflammatory mediators in RA^10^. Additionally, Croasdell et al. found that RvD2 induced miR-146a to reduce TLR4 expression in human monocytes^22^. Serhan proposed that SPMs might also regulate microRNA in novel resolution circuits in inflammation^23^. Moreover, Neudecker’s and Zhang’s teams simultaneously found that miR-233 repressed NLRP3 inflammasome to regulate the innate immune responses^24,25^. Additionally, Li XF et al. reported that miR-20a negatively regulated expression of NLRP3-inflammasome by targeting thioredoxin-interacting protein (TXNIP) in adjuvant-induced arthritis (AA) fibroblast-like synoviocytes (FLS)^26^. Meanwhile, miR-23a, miR-21, and miR-200a were also confirmed to target the TXNIP/NLRP3 axis to regulate chronic inflammatory responses^27–29^. Therefore, we used a miRNA microarray to screen different expression levels of miRNAs in bone marrow-derived macrophages (BMDMs) after PDX treatment and to explore these miRNAs’ role in the regulation of PDX in the NLRP3 signaling pathway during RA pathogenesis.

In this study, we also analyzed the expression of PDX in serum from active- or inactive-RA patients and investigated the effect of PDX in RA and the exact mechanism thereof.

## Results

### PDX levels decreased in active-RA patients and increased in inactive-RA patients

**Fig. 1:**
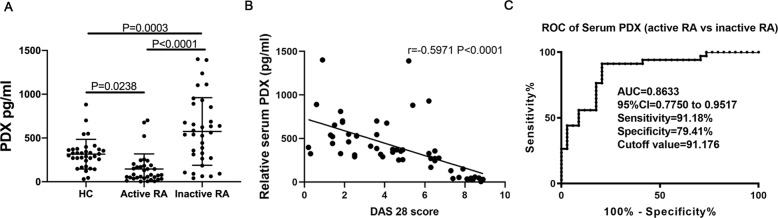
Concentrations of PDX in active- and inactive-RA patients and in HCs. **A** Levels of PDX in serum samples from HCs (*n* = 34), patients with inactive RA (*n* = 34), and patients with active RA (*n* = 34; F[3,102] = 22.97). **B** Correlation between serum PDX level and DAS28 score (*n* = 54). **C** ROC curve analysis was performed to analyze the diagnostic value of serum PDX expression levels in active RA. Data were presented as means ± SD. Differences among the three groups were assessed by one-way ANOVA, and *post hoc* tests (Tukey’s method) were applied to investigate the differences one by one.

We detected levels of PDX in serum using UPLC-MS/MS. They were higher in inactive-RA patients and lower in active-RA patients than in HCs (Fig. 1A, Supp. Fig. S1). Consistent with these findings, we observed an inverse correlation between serum PDX level and DAS28 (*r* = −0.59, *P* < 0.0001; Fig. 1B). Moreover, we analyzed the diagnostic value of serum PDX for active RA using ROC curve analysis. Sensitivity, specificity and area under the curve (AUC) were respectively 0.9118, 0.7941, and 0.8633 (95% confidence interval [CI], 0.7750–0.9517) at a cutoff value of 91.176 pg/mL (Fig. 1C).

### PDX attenuated joint injury and inflammatory response in the CIA model

**Fig. 2:**
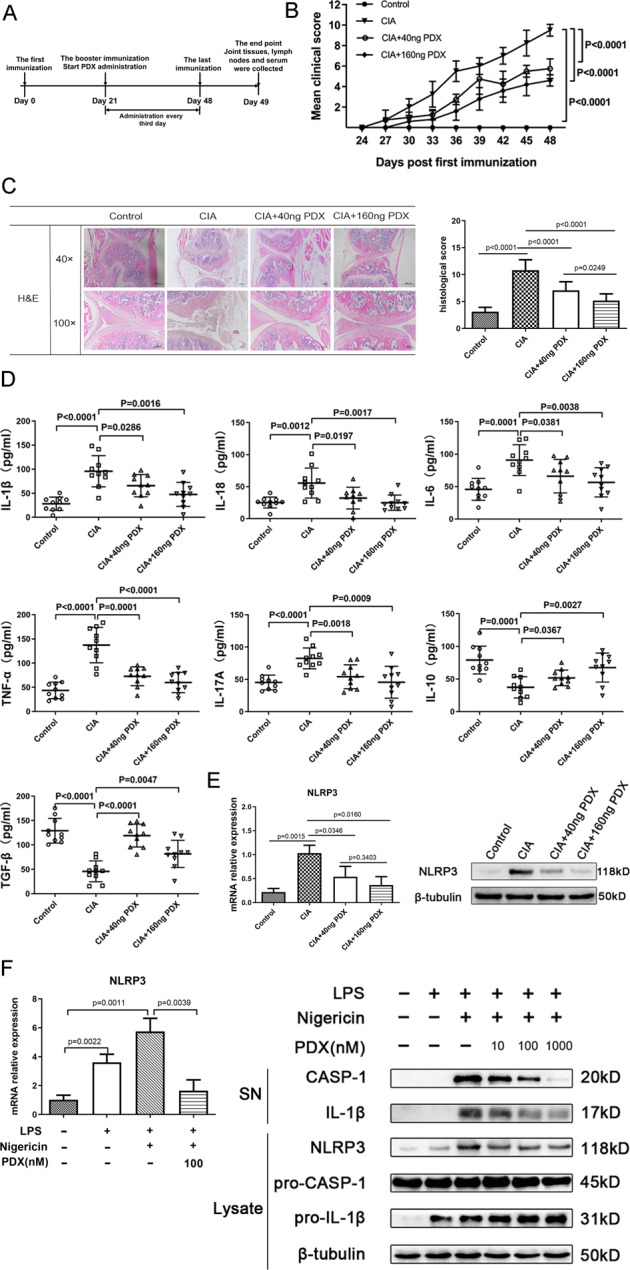
Effect of PDX on joint destruction and inflammatory response in CIA models and LPS-activated BMDMs. **A** Timeline of PDX treatment experiment in the CIA model. **B** Clinical scores of CIA mice treated or not treated with PDX. Statistical significance was defined by ANOVA of repeated measurements. **C** H&E staining of knee joints of CIA mice (40×, 100×) and histopathological analysis of H&E staining (F[2,30] = 34.69). **D** Concentrations of serum cytokines IL-1β, IL-18, IL-6, TNF-α, IL-17A, IL-10, and TGF-β in PDX-treated CIA mice as shown by ELISA. **E** Expression of NLRP3 in PDX-treated CIA mice as shown by RT-qPCR and WB. **F** Expression of NLRP3 mRNA, NLRP3 protein, pro–CASP-1, pro–IL-1β in lysate, and CASP-1 and IL-1β in supernatant of LPS-activated BMDMs as shown by RT-qPCR and WB. SN = supernatant. Data were presented with means ± SD. The differences of C–I were assessed by one-way ANOVA and the post hoc tests (Turkey method) were applied to investigate the differences one by one.

We constructed CIA model to confirm the effect of PDX on RA progression. Results showed that PDX obviously reduced clinical scores and paw swelling (Fig. 2A, B), as well as histological score (Fig. 2C), in CIA mice dose-dependently. Furthermore, it inhibited pro-inflammatory cytokines IL-1β, IL-18, IL-6, TNF-α and IL-17A while facilitating anti-inflammatory cytokines IL-10 and TGF-β, also dose-dependently (Fig. 2D). Based on the above findings, we proved for the first time that PDX could ameliorate joint damage and suppress inflammation in RA. Moreover, mRNA and protein levels of NLRP3 were reduced in lymph nodes of PDX-treated CIA mice (Fig. 2E). Therefore, we performed a PDX intervention experiment in LPS-activated BMDMs. As expected, PDX reduced mRNA and protein levels of NLRP3 in vitro, as well as levels of NLRP3 inflammasome–related proteins IL-1β and CASP-1 (Fig. 2F). These findings suggested that PDX effectively inhibited inflammatory responses and restrained the NLRP3 signaling pathway.

### PDX restored Treg/T_h_17 cell balance in the CIA model

**Fig. 3:**
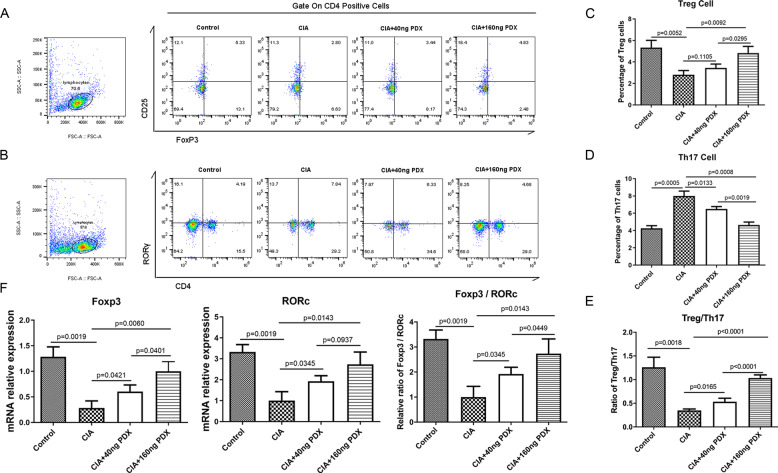
PDX regulated Treg/Th17 cell balance in CIA mice. **A–D** Percentages of T_h_17 cells and Tregs from PDX-treated CIA mouse lymph nodes as shown by FCM (F[4, 12] = 61.37 and F[4,12] = 14). **E** Ratio of Tregs to T_h_17 cells from PDX-treated CIA mice (F[4,12] = 72.83). **F** mRNA expression of RORγ and FOXP3, and FOXP3/RORγ ratio, in lymphocytes from PDX-treated CIA mice, as shown by RT-qPCR (F[4, 12] = 16.86 for RORγ, F[4,12] = 21.32 for FOXP3 and F[4,12] = 19.56 for FOXP3/RORγ). Data were presented as means ± SD. Differences among the three groups were assessed using one-way ANOVA, and *post hoc* tests (Tukey’s method) were applied to investigate the differences one by one.

No study has ever shown the efficacy of PDX intervention on the balance of Tregs and T_h_17 cells. Ours was the first to discover that PDX increased the proportion of Tregs to T_h_17 cells and the mRNA level of the Treg-characteristic transcription factor, forkhead box P3 (FOXP3), also dose-dependently (Fig. 3A, C). By contrast, PDX decreased the proportion of T_h_17 cells to Tregs and the mRNA level of the T_h_17 cell–characteristic transcription factor, retinoic acid receptor (RAR)–related orphan receptor gamma (RORγ), in a dose-dependent manner (Fig. 3B, D). Consequently, the PDX-treated group showed elevated Treg/T_h_17 cell and FOXP3/RORγ ratios compared with non-PDX-treated group (Fig. 3E, F). Having determined these points, we identified for the first time that PDX could restore Treg/T_h_17 cell balance, which was disordered during CIA construction.

### PDX attenuated joint injury and inflammatory response via the NLRP3 signaling pathway

**Fig. 4:**
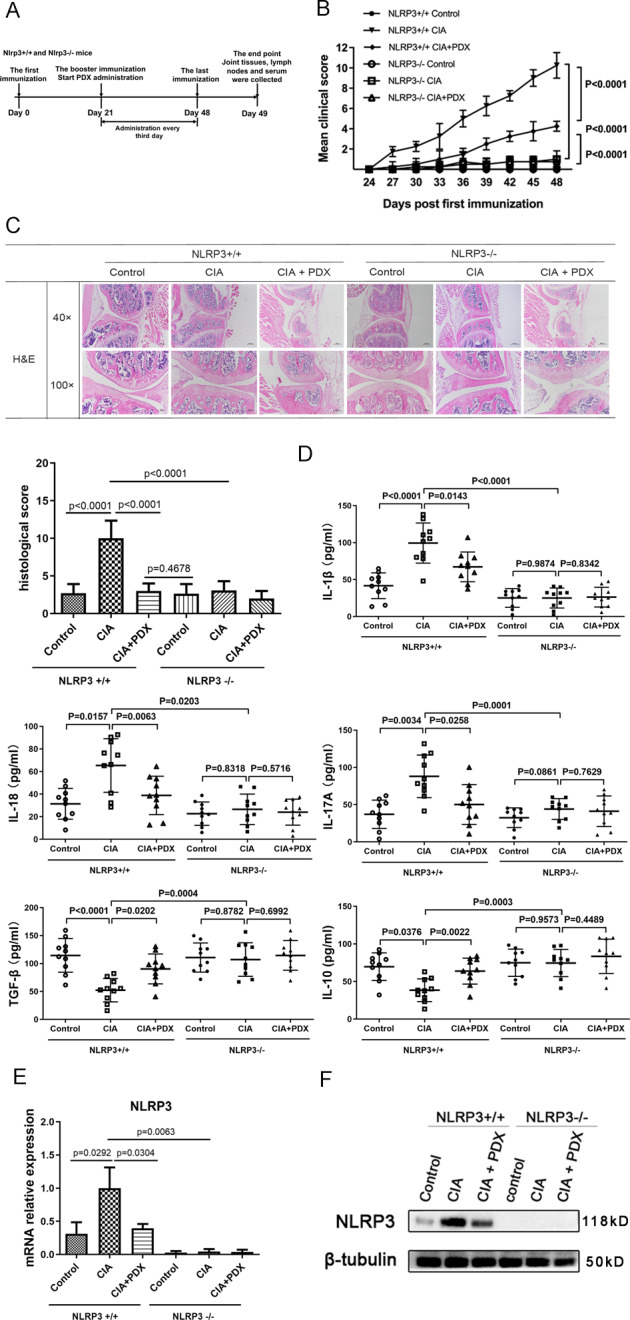
Effect of PDX in alleviating inflammation and joint destruction in Nlrp3^−/−^ CIA mice. **A** Timeline of PDX treatment experiment in *Nlrp*3^+/+^ and *Nlrp*3^−/−^ mouse CIA models. **B** Clinical scores of PDX-treated CIA models established using *Nlrp*3^+/+^ and *Nlrp*3^−/−^ CIA mice. Statistical significance was defined by ANOVA of repeated measurements. **C** H&E staining of knee joints from PDX-treated *Nlrp*3^+/+^ and *Nlrp*3^−/−^ CIA mice (40×, 100×) (upper panel) and histopathological analysis of H&E staining (F[3,40] = 54.02) (lower panel). **D** Concentrations of serum cytokines IL-1β, IL-18, IL-17A, IL-10, and TGF-β in PDX-treated *Nlrp*3^+/+^ and *Nlrp*3^−/−^ CIA mice as shown by ELISA. **E** Expression of NLRP3 mRNA in lymphocytes from PDX-treated *Nlrp*3^+/+^ and *Nlrp*3^−/−^ CIA mice as shown by RT-qPCR (F[6,18] = 18.62). **F** NLRP3 protein levels in lymph nodes of PDX-treated *Nlrp*3^+/+^ and *Nlrp*3^−/−^ CIA mice as shown by WB.

We used *Nlrp*3^−/−^ mice to verify the mechanism by which PDX attenuated RA progression. First, we verified the successful knockout of *Nlrp*3 using RT-qPCR and WB (Fig. 4E, F). Next, we constructed CIA models. Clinical symptoms of *Nlrp*3^−/−^ mice were obviously milder than those of *Nlrp*3^+/+^ mice (Fig. 4A, B). On the one hand, PDX intervention obviously postponed onset of RA and reduced histological scores in *Nlrp*3^+/+^ mice compared with *Nlrp*3^−/−^ mice (Fig. 4C). On the other hand, after *Nlrp*3 knockout, levels of pro-inflammatory cytokines IL-1β, IL-18, IL-17A decreased, while those of anti-inflammatory cytokines IL-10 and TGF-β increased substantially in the serum of CIA mice (Fig. 4D). Simultaneously, PDX intervention suppressed inflammatory responses in *Nlrp*3^+/+^ mice, but much weaker efficacy was noted in *Nlrp*3^−/−^ mice. These results suggested that PDX resolved inflammation mainly through the NLRP3 signaling pathway.

### PDX restored Treg/T_h_17 cell balance via the NLRP3 signaling pathway

**Fig. 5:**
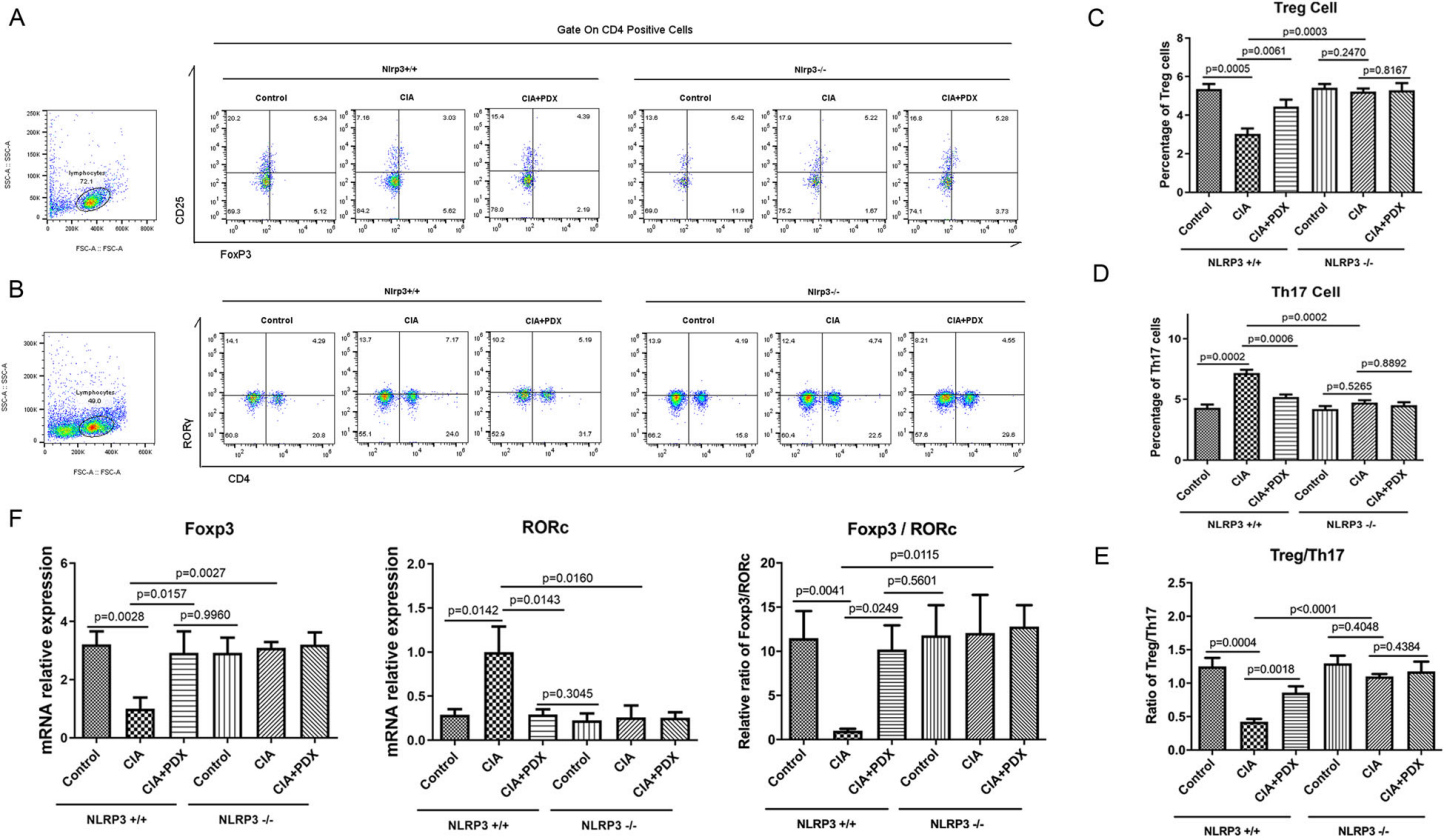
Effect of PDX in restoring Treg/Th17 cell balance in Nlrp3^−/−^ CIA mice. **A–D** Percentages of T_h_17 cells and Tregs in PDX-treated *Nlrp*3^+/+^ and *Nlrp*3^−/−^ CIA mice as shown by FCM (respectively, F[6,18] = 61.58 and F[6,18] = 31.29). **E** Ratio of Tregs to T_h_17 cells in PDX-treated *Nlrp*3^+/+^ and *Nlrp*3^−/−^ CIA mice (F[6,18] = 31.70). **F** mRNA expression of RORγ and FOXP3, and FOXP3/RORγ ratio, in lymphocytes from PDX-treated *Nlrp*3^+/+^ and *Nlrp*3^−/−^ CIA mouse lymph nodes as shown by RT-qPCR (F[6,18] = 13.62 for RORγ, F[6,18] = 9.584 for FOXP3 and F[6,18] = 37.08 for FOXP3/RORγ). Data were presented as means ± SD. Differences among the three groups were assessed by one-way ANOVA, and *post hoc* tests (Tukey’s method) were applied to investigate the differences one by one.

To further explore the key effect of the NLRP3 signaling pathway on PDX’s restoration of Treg/T_h_17 cell balance, we treated *Nlrp*3^−/−^ CIA mice with PDX. Compared with the control group, PDX intervention led to a decrease in the proportion of T_h_17 cells and an increase in that of Tregs in lymph nodes from *Nlrp*3^+/+^ mice, while no significant change was detected in lymph nodes from *Nlrp*3^−/−^ mice (Fig. 5A–D). Not surprisingly, Treg/T_h_17 cell ratios were elevated in PDX-treated *Nlrp*3^+/+^ mice but not in *Nlrp*3^−/−^ mice (Fig. 5E). Simultaneously, PDX reduced mRNA levels of RORγ, increased mRNA levels of FOXP3, and elevated FOXP3/RORγ ratios in lymph nodes from *Nlrp*3^+/+^ mice, while these changes were not found in *Nlrp*3^−/−^ mice (Fig. 5F). All of the above demonstrated that PDX could restore Treg/T_h_17 cell balance in CIA mice, a process mediated by NLRP3.

### PDX restored Treg/T_h_17 cell balance in a rescue experiment involving Nlrp3^−/−^ CIA mice

**Fig. 6:**
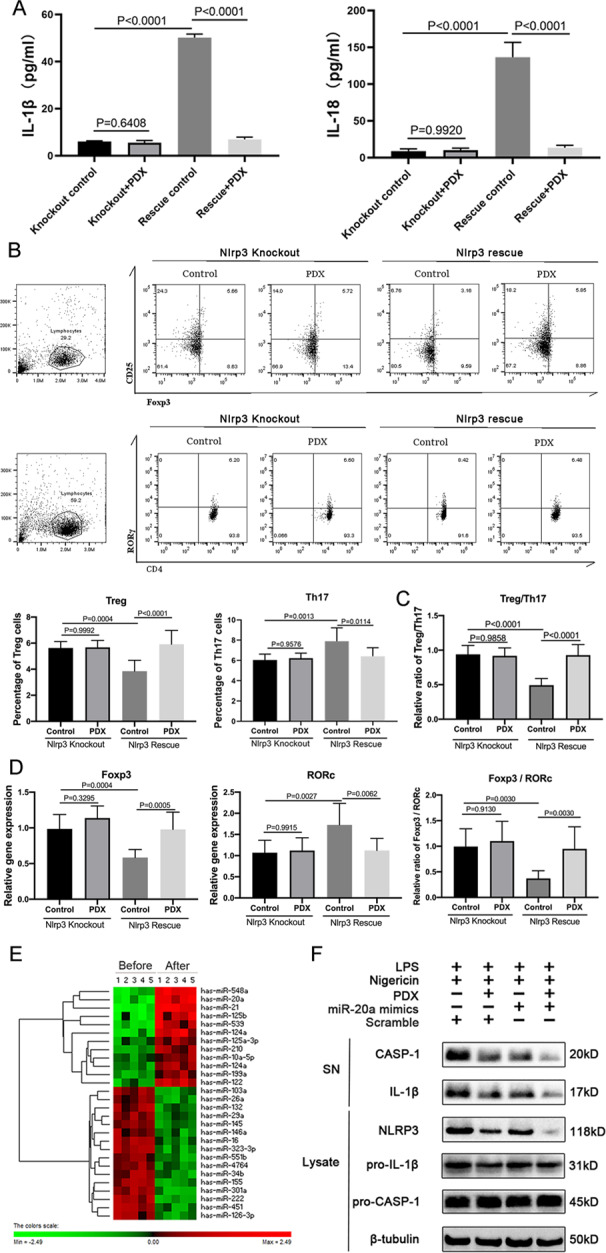
PDX, regulated by miR-20a, restored Treg/Th17 cell balance in a rescue experiment involving Nlrp3^−/−^ CIA mice. **A** Concentrations of IL-1β and IL-18 were detected in supernatant via ELISA. **B** Naive T cells were isolated and used as intervention in supernatant of PDX-treated BMDMs from NLRP3-rescued *Nlrp*3^−/−^ CIA mice. Percentages of T_h_17 cells and Tregs differentiated from naive T cells were detected via FCM. **C** Ratio of Tregs to T_h_17 cells in the rescue experiment. **D** RORγ and FOXP3 mRNA expression and FOXP3/RORγ ratio. **E** Heatmap analysis of different miRNA expression profiles in macrophages before and after PDX microarray treatment. **F** Concentrations of CASP-1, IL-1β, NLRP3, pro–CASP-1, and pro–IL-1β in miR-20a–transfected macrophages were detected via WB.

We conducted a NLRP3 rescue experiment to explore the effect of NLRP3 on PDX-induced differentiation of CD4^+^ T cells. Intervention with supernatant from PDX-treated BMDMs from *Nlrp*3^−/−^ CIA mice showed no significant difference in the proportion of T_h_17 cells to Tregs, compared with supernatant from controls. Furthermore, rescue of NLRP3 increased the proportion of T_h_17 cells and decreased that of Tregs. PDX then restored Treg/T_h_17 cell balance, which was disordered after NLRP3 rescue (Fig. 6B, C). It also attenuated inflammatory aggravation caused by NLRP3 rescue, a finding revealed by a decrease in RORγ mRNA, IL-1β, and IL-18 expression and an increase in FOXP3 mRNA expression (Fig. 6A, D).

### PDX restored Treg/T_h_17 cell balance by inhibiting NLRP3 inflammasome via miR-20a

To investigate the mechanism by which PDX reduced NLRP3 expression and ultimately restored Treg/T_h_17 cell balance in RA, we analyzed expression profiles of miRNAs using a microarray of BMDMs treated with PDX (Fig. 6E). Based on screening and verification of different miRNAs, we selected overexpressed miR-20a for further study (Supp. Fig. S2). Overexpression of miR-20a reduced NLRP3, CASP-1, and IL-1β, which was particularly obvious with PDX intervention (Fig. 6F). In conclusion, PDX inhibited NLRP3 expression via regulation of miR-20a in RA.

## Discussion

RA is a chronic, systemic inflammatory autoimmune disease characterized by progressive articular damage and functional loss^1^. Inflammatory response plays an important role in the pathogenesis of RA and is suggested as key to therapy for the disease^30^. The function of SPMs in inflammation resolution in RA has attracted tremendous attention in recent years. Our previous studies have confirmed that MaR1 and RvD1 can effectively ameliorate RA progression^10,11^. Meanwhile, protectin D1 is a classic resolution activator in acute inflammation that regulates leukocyte infiltration^31^. However, the role of PDX, the isomer of protectin D1, in the progression of RA is still unclear. Herein, we are the first to report a decrease in PDX in patients with active RA and an increase in PDX in those with inactive RA compared with HCs, and that serum PDX levels correlated negatively with DAS28. Furthermore, we innovatively demonstrated that serum PDX was increased in inactive-RA cases and reduced in active-RA cases compared with HCs, which indicated that PDX might be an important bio-index for detecting RA activity. These facts suggested that disordered PDX levels could be an important factor in RA pathogenesis and that a decline of PDX in RA patients might play a vital role in converting the inactive phase into the active phase. Moreover, exogenously upregulating PDX in active-RA patients might be a promising avenue of treatment.

To verify the effect of PDX in the progression of RA and provide statistics for clinical research, we first explored the effect of PDX in RA pathogenesis. As expected, PDX intervention obviously delayed CIA progression; joint H&E staining showed mitigation of cartilage and bone damage. Meanwhile, serum cytokine expression as measured by ELISA showed remission of inflammatory response. These results suggested a protective effect of PDX in the pathogenesis of RA.

Although the disease’s precise pathogenesis remains unclear, T-cell differentiation is reported to participate in the process^32^. Furthermore, abundant research indicates that Treg/T_h_17 cell imbalance is one sign of RA pathogenesis^7,8^. However, although it is known as a regulator of M1/M2 macrophage differentiation in sepsis, PDX’s effect on the regulation of T-cell differentiation in RA has still not been studied^33^. Our previous study and that by Ruiz A et al. reported that RvD1, RvD2, and MaR1 participate in modulating T-cell response, suggesting a new avenue for SPM-based approaches to modulating chronic inflammation^11,34^. Therefore, in this study, we innovatively detected percentages of both Tregs and T_h_17 cells in PDX-treated lymph nodes of CIA mice using FCM. Results showed a higher proportion of Tregs and a lower proportion of T_h_17 cells, revealing that PDX could restore Treg/T_h_17 cell balance. Additionally, an inverse correlation between FOXP3/RORγ ratio and clinical score was validated in CIA mice, suggesting that PDX participated in RA pathogenesis by restoring Treg/T_h_17 cell balance. At the same time, NLRP3 and the CASP-1/IL-1β axis decreased in lymph nodes of the PDX intervention CIA group, as shown by RT-qPCR and WB. Therefore, we went on to explore the mechanism by which PDX restored Treg/T_h_17 cell balance and the function of the NLRP3 inflammasome pathway in this process.

NLRP3 inflammasome is a complex of NLRP3, ASC, and CASP-1 that activates CASP-1 to induce IL-1β, TNF-α, and other cytokines^35^. Recent studies confirm that NLRP3 inflammasome regulates T_h_17 cell differentiation in RA, but the influence of NLRP3 on Treg/T_h_17 cell balance is still seldom mentioned^36^. Considering the influence of SPMs on NLRP3, Aritz et al. found that RvD2 (an SPM) blocked NLRP3 inflammasome, leading to reduced release of IL-1β into exudates of macrophages. This suggests that SPMs can inhibit the priming and expedite the deactivation of NLRP3 inflammasome in acute inflammation^37^. In this study, we innovatively detected the effect of PDX on NLRP3 inflammasome activation in chronic inflammation. Results revealed that PDX reduced NLRP3 expression and inhibited activation of the CASP-1/IL-1β axis. To further verify these results, we used *Nlrp*3^−/−^ mice to construct a CIA model, rescued the mice with NLRP3, and intervened with PDX. As expected, pro-inflammatory cytokines were decreased, anti-inflammatory cytokines were increased and Treg/T_h_17 cell ratio was increased in *Nlrp*3^−/−^ mice compared with *Nlrp*3^+/+^ mice. Interestingly, PDX almost lost its inflammatory-suppression function in *Nlrp*3^−/−^ mice. Furthermore, NLRP3 rescue in vitro showed obvious aggravation of inflammation and Treg/T_h_17 cell imbalance. PDX resolved inflammatory response and restored Treg/T_h_17 cell balance, which further verified its ability to attenuate CIA pathogenesis by inhibiting the NLRP3 inflammasome pathway. Based on these findings, in this article, we suggest that PDX regulates Treg/T_h_17 cell balance by inhibiting NLRP3 inflammasome in RA, which still needs further confirmation on humans.

In recent studies, miRNAs have emerged as key regulators in the process by which SPMs exercise biological functions^37^. Our previous study found that miR-21 is a key factor in the mechanism by which MaR1 restores Treg/T_h_17 cell balance in RA patients^11^. Additionally, Wu YH et al. recently found that miR-16 might be implicated in Treg/T_h_17 cell imbalance in RA patients^38^. Therefore, we performed miRNA microarray studies in macrophages treated with PDX. Based on screening and RT-qPCR verification, we selected miR-20a for further study. MiR-20a has been shown to downregulate the NLRP3 inflammasome in AA FLS^26^. Additionally, miR-20a has also been reported to downregulate the mitogen-activated protein kinase 1 (*MAPK1*) signaling pathway to alter cell proliferation and differentiation, including T_h_1 and T_h_17 cell reduction and Treg induction^39^. Xie Z et al. confirmed that miR-20a is downregulated in RA tissues and that it downregulates fibroblast-like synoviocyte proliferation and apoptosis^40^. Therefore, we performed miR-20a intervention in BMDMs from CIA mice. Results showed that NLRP3 expression tended to be inhibited in BMDMs transfected with miR-20a, particularly obvious with PDX intervention along, demonstrating that miR-20a participated in the mechanism by which PDX inhibited the NLRP3 inflammasome pathway to restore Treg/T_h_17 cell balance in RA. Additionally, overexpression of PDX alone showed a mildly stronger effect on NLRP3 down-regulation than miR-20a alone. In consideration that miR-20a is overexpressed with the treatment of PDX, we reckoned PDX can reduce the expression of NLRP3, may not only through miR-20a, but also in other pathways.

In summary, we innovatively confirmed that PDX could effectively ameliorate CIA progression by restoring Treg/T_h_17 cell balance, which was mediated by miR-20a’s inhibition of the NLRP3 inflammasome pathway via miR-20a.

## Methods and materials

### Patients and samples

We obtained blood samples from RA patients and HCs to examine PDX levels. Serum from active-RA (Disease Activity Score 28-joint assessment [DAS28] > 3.2) and inactive-RA (DAS28 < 3.2) groups was acquired on the first day of clinical admission prior to commencement of any treatment during current hospitalization (bDMARDs-naive). All samples were collected at the First Affiliated Hospital of Wenzhou Medical University (WMU; Wenzhou, China). We based RA diagnosis on 2010 American College of Rheumatology (ACR) criteria. Samples were extracted with patients’ informed consent and have passed the review of the First Affiliated Hospital of Wenzhou Medical University Ethics Committee. Elaborate clinical details are provided in Supp. Table S1.

### UPLC-MS/MS-based PDX determination

Concentrations of PDX (10[S], 17[S]-dihydroxy-4Z,7Z,11E,13Z,15E,19Z- docosahexaenoic acid; Chemical Abstracts Service [CAS] No. 871826-47-0; Cayman Chemical Co., Ann Arbor, MI, USA) in human serum samples were assessed as described previously^23,41^. We extracted samples (2 mL each) using ice-cold methanol (4 mL). Prior to extraction, we added 500 pg d4-LTB4 (5[S], 12[R]-dihydroxy-6Z, 8E, 10E, 14Z-eicosatetraenoic-6, 7, 14, 15-d4 acid; CAS No. 124629-74-9; Cayman Chemical) as an internal standard to improve the accuracy and precision of the assay. Lipid mediators (LMs) were extracted by solid-phase extraction (SPE) using a C18 SPE cartridge. We analyzed methyl formate fractions for PDX levels via ultraperformance liquid chromatography-tandem mass spectrometry (UP LC-MS/MS) using a UPLC I-Class system (Waters Corp., Milford, MA, USA) equipped with an Agilent Eclipse Plus C18 column (2.1 mm × 100 mm × 1.7 µm; Agilent Technologies, Inc., Santa Clara, CA, USA) paired with a SCIEX 6500 Q-TRAP mass spectrometer (SCIEX, Framingham, MA, USA). Instrument control and data acquisition were performed using Analyst software version 1.6.2 (SCIEX). To monitor and quantify levels of the various LMs, we developed a selected-reaction monitoring (SRM) method with signature ion fragments for PDX. Identification was based on previously published criteria wherein a minimum of four diagnostic ions were employed. We performed quantification based on peak area ratio of PDX to internal standard d4-LTB4 in SRM transitions and the linear calibration curve of PDX. The procedure mentioned above refers to the method described by our previous study^11^.

### Collagen-induced arthritis (CIA) mouse model construction and clinical evaluation

We purchased 8-week-old Dilute, Brown, and non-Agouti 1 (DBA/1) mice weighing 18–20 g from Shanghai Laboratory Animal Center (SLAC; Shanghai, China). *Nlrp*3^−/−^ mice were purchased from Zhejiang University Laboratory Animal Center (ZJULAC; Zhejiang, China). All procedures in the animal experiments were endorsed by the Institutional Animal Care and Use Committee of WMU. All mice were raised in a specific-pathogen-free (SPF) room at the Laboratory Animal Center of WMU, housed in groups of five per cage and kept at room temperature (RT; 22–26 °C) and 60–65% humidity on a regular 12-h light/dark cycle (light, 8:30–20:30). The person raising the mice routinely monitored their health status and observed no adverse events.

We intradermally induced autoimmune arthritis by injecting a 100-μL emulsion of complete Freund’s adjuvant and type II bovine collagen (2 mg/mL; both from Chondrex, Redmond, WA, USA) into the mice’s backs on day 0. A 100-μL shot of type II bovine collagen (2 mg/mL) emulsified with incomplete Freund’s adjuvant (Chondrex) was given as a booster 3 weeks later. Mice were then treated with PDX (Cayman Chemical; 0, 40, or 160 ng per mouse, *n* = 10 per group) by tail vein injection every 3 days until day 48, and were sacrificed on day 49. Two individuals who were ignorant of the animals’ treatment observed the mice for 3 days for arthritis severity. Scoring was as follows: 0 = no evidence of erythema or swelling; 1 = erythema and mild swelling confined to the tarsals or ankle joint; 2 = erythema and mild swelling extending from the ankle to the tarsals; 3 = erythema and moderate swelling extending from the ankle to metatarsal joints; and 4 = erythema and severe swelling encompassing the ankle, paw, and digits, or ankylosis of the limb. We harvested joint tissues, lymph nodes, and serum for further research. We prepared lymph nodes for reverse-transcription quantitative real-time polymerase chain reaction (RT-qPCR), Western blot (WB), and flow cytometry (FCM). Serum was prepared for an enzyme-linked immunosorbent assay (ELISA). Limbs were fixed in 4% paraformaldehyde, decalcified in 50 nM ethylenediaminetetraacetic acid (EDTA) solution, and embedded in paraffin.

### Histological analysis

We fixed the knee joints in 4% paraformaldehyde, decalcified them in 50 nM EDTA and embedded them in paraffin. Sections were then deparaffinized, rehydrated, and stained with H&E. We semiquantitatively scored them for inflammatory-cell infiltration, synovial hyperplasia, and bone destruction using H&E staining and then graded them on a scale of 0 (normal) to 3 (severe) for 4 paws in 12 totally.

### Enzyme-linked immunosorbent assay (ELISA)

Levels of IL-1β, IL-18, TGF-β, IL-10, IL-17A, IL-6, and TNF-α in serum were detected by ELISA. We purchased antibodies of these cytokines from BioLegend CNS, Inc. (San Diego, CA, USA). Specimens were diluted to 50 μL (1:20) per manufacturer’s instructions and measured at an optical density (OD) of 450 nm.

### Western blot (WB)

We prepared lysates and isolated proteins using sodium dodecyl sulfate polyacrylamide gel electrophoresis (SDS-PAGE) and transferred them onto polyvinylidene difluoride (PVDF) membranes. Blots were incubated with primary antibodies (anti-NLRP3 [AdipoGen Life Sciences, San Diego, CA, USA], anti–CASP-1 [R&D Systems, Inc., Minneapolis, MN, USA], anti–IL-1β [Santa Cruz Biotechnology, Dallas, TX, USA] or β-tubulin [bioWORLD, Dublin, OH, USA]) overnight at 4 °C. Incubation with secondary anti-goat–horseradish peroxidase (HRP) and anti-rabbit–HRP (Hangzhou MultiSciences [Lianke] Biotech Co., Ltd., Hangzhou, China) was performed at RT for 1 h. We used an ECL Plus Western Blot Detection Kit (Thermo Fisher Scientific, Waltham, MA, USA) for antibody detection.

### Flow cytometry (FCM)

We harvested inguinal lymph nodes from sacrificed CIA mice, triturated them with a 300-mesh cell strainer, gently washed them twice in PBS, and then resuspended them in Roswell Park Memorial Institute (RPMI) 1640 medium. To assess T_h_17 cells and Tregs, we stimulated lymphocytes for 4 h with phorbol myristate acetate (25 ng/mL), ionomycin (1 µg/mL), and brefeldin A (10 µg/mL) and then stained them with APC–conjugated anti-CD4 and fluorescein isothiocyanate (FITC)–conjugated anti-CD25 for 30 min at 4 °C in the dark. After surface staining, we then respectively labeled T_h_17 cells and Tregs with phycoerythrin (PE)–conjugated anti-RORγ and PE-conjugated anti-FOXP3 for 30 min at 4 °C in the dark. FCM was performed on a Cytoflex system (BD Biosciences, Franklin Lakes, NJ, USA) and analyzed using FlowJo software (FlowJo LLC [BD Biosciences]). All reagents involved in this experiment were purchased from BD Biosciences.

### RNA isolation, miRNA microarray, and RT-qPCR

Total RNA samples (both tissues and cells) were isolated from mice using TRIzol reagent (Ambion, Inc., Austin, Texas, US) per manufacturer’s protocol. We determined miRNA expression profiles of BMDMs treated with 10 nM PDX using miRNA microarray analysis. We reverse-transcribed the complementary deoxyribonucleic acid (cDNA) of mRNA (1 μg) and of the miRNA itself using a PrimeScript RT Reagent Kit (TaKaRa Bio, Shiga, Japan) and measured the expression of the target gene using a SYBR Fast Universal qPCR Kit (Kapa Biosystems, Inc. [Roche Life Science, Basel, Switzerland]) for RT-qPCR. Primer sequences used are listed in online Supp. Table S2. We normalized miRNAs to U6 controls and mRNAs to β-actin; conversion was performed using relative quantification (2^–ΔΔCt^).

### Mouse naive T-cell and bone marrow-derived macrophage (BMDM) purification and cultivation, PDX intervention, and NLRP3 rescue experiment

We collected inguinal lymph nodes of DBA/1 mice and pooled them for cell extraction using a complete RPMI 1640 medium. T cells were positively isolated with anti-CD4–coated magnetic beads. The purity of naive CD4^+^ T-cells was >95%, as confirmed by FCM. The cells were then harvested and activated by plate-bound anti-CD3 (5 µg/mL) and anti-CD28 (2 µg/mL) (Biolend, California, USA).

We rinsed bone marrow cells obtained from bilateral hind femurs of DBA/1 mice in RPMI 1640 medium. Then, we cultured 1 × 10^6^ BMDMs from *Nlrp*3^+/+^ mice and *Nlrp*3^−/−^ mice in, respectively, RPMI 1640 medium supplemented with 10% of heat-inactivated fetal bovine serum (FBS) and 2 mM L-glutamine/20% conditioned L929 medium for 6 days. Adherent macrophages were harvested and stimulated with LPS (6 h) and nigericin (2 h) to activate NLRP3 inflammasome in vitro and then treated with PDX (10, 100, or 1000 nM) for 2 h. We obtained and used the supernatant for naive CD4^+^ T-cell intervention for 7 days. IL-1β (10 ng/mL), IL-6 (20 ng/mL), IL-23 (100 ng/mL) and TGF-β (1 ng/mL) were added for Th17 cells polarization, while TGF-β (2 ng/mL) and IL-2 (20 U/mL) (Miltenyi Biotec, Germany) were added for Treg cells polarization.

The GV309 lentivirus vector (Genechem, China) was used to upregulate NLRP3 expression in BMDMs from *Nlrp*3^−/−^ mice. The assays of lentivirus infection were performed according to manufacturer’s instructions. Briefly, 50 µL GV309 lentivirus vector and 50 µL poly-brene (50 µg/mL; Sigma-Aldrich) were added in a 24-well plate containing 500 µL RPMI 1640 and 1 × 10^5^ BMDMs. After 6 hours, the transfection medium was replaced by 500 µL RPMI 1640 containing 10% fetal bovine serum.

### Statistical analysis

Data were analyzed using SPSS software version 22.0 (IBM Corp., Armonk, NY, USA) and GraphPad Prism version 8 (GraphPad Software, Inc., San Diego, CA, USA). We used the Shapiro–Wilk method to judge normally distributed data and the Levene method to test homogeneity of variance. Two sets of data that met normal distribution and homogeneity of variance were analyzed using Student’s *t* test. Multigroup comparisons of means were performed using a one-way analysis of variance (ANOVA) test, with post hoc contrasts performed using Tukey’s test. The rest of the data that did not meet homogeneity of variance or normal distribution was compared using Kruskal–Wallis and Mann–Whitney non-parametric tests. We evaluated the discriminatory ability of PDX in active RA using receiver operating characteristic (ROC) curve analysis, with active-RA cases as true-positive cases and inactive-RA cases as true-negative cases. *P*-values < 0.05 were considered significant.

## Supplementary information

Figure S1. MS/MS spectrum of PDX.

Figure S2. (A-F) The expression of miR-20a, miR-539, miR-125b, miR-26a, miR-124a and miR-145 in BMDMs treated with PDX was verified by RT-qPCR.

Table S1 Characteristics of patients with active RA, inactive RA and healthy controls.

Table S2. Sequence(5’-3’) of primers for RT-qPCR and miRNA microarray.
