# Supplementary figures and images for: Protectin DX restores Treg/Th17 cell balance in rheumatoid arthritis by inhibiting NLRP3 inflammasome via miR-20a
Source: Cell Death Dis. 2021 Mar 15;12(3):280. doi: 10.1038/s41419-021-03562-6 (PMC7961047; doi:10.1038/s41419-021-03562-6)

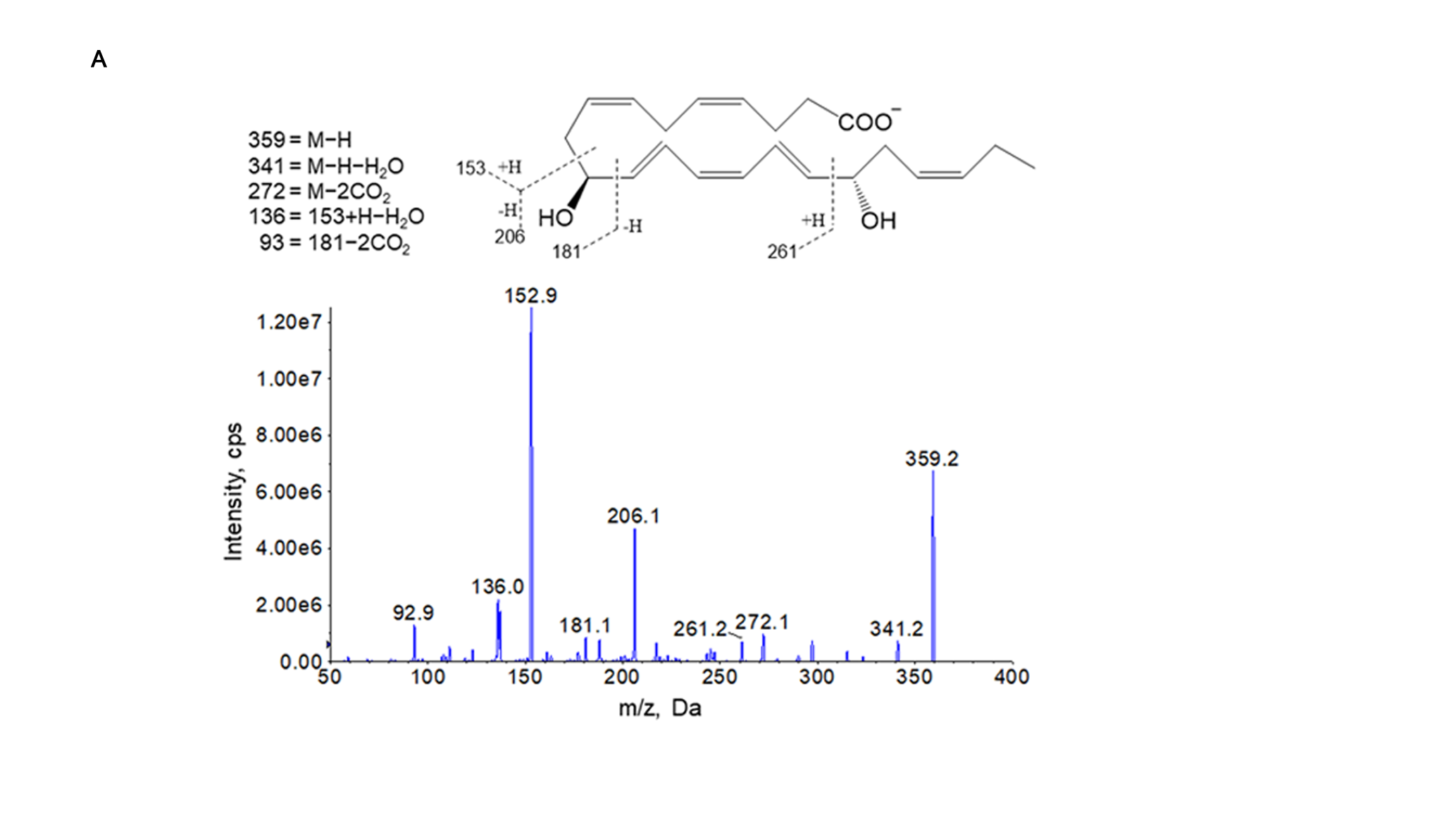

Supplement: Supplementary file 1 — Figure S1. MS/MS spectrum of PDX. [file 41419_2021_3562_MOESM1_ESM.tif]

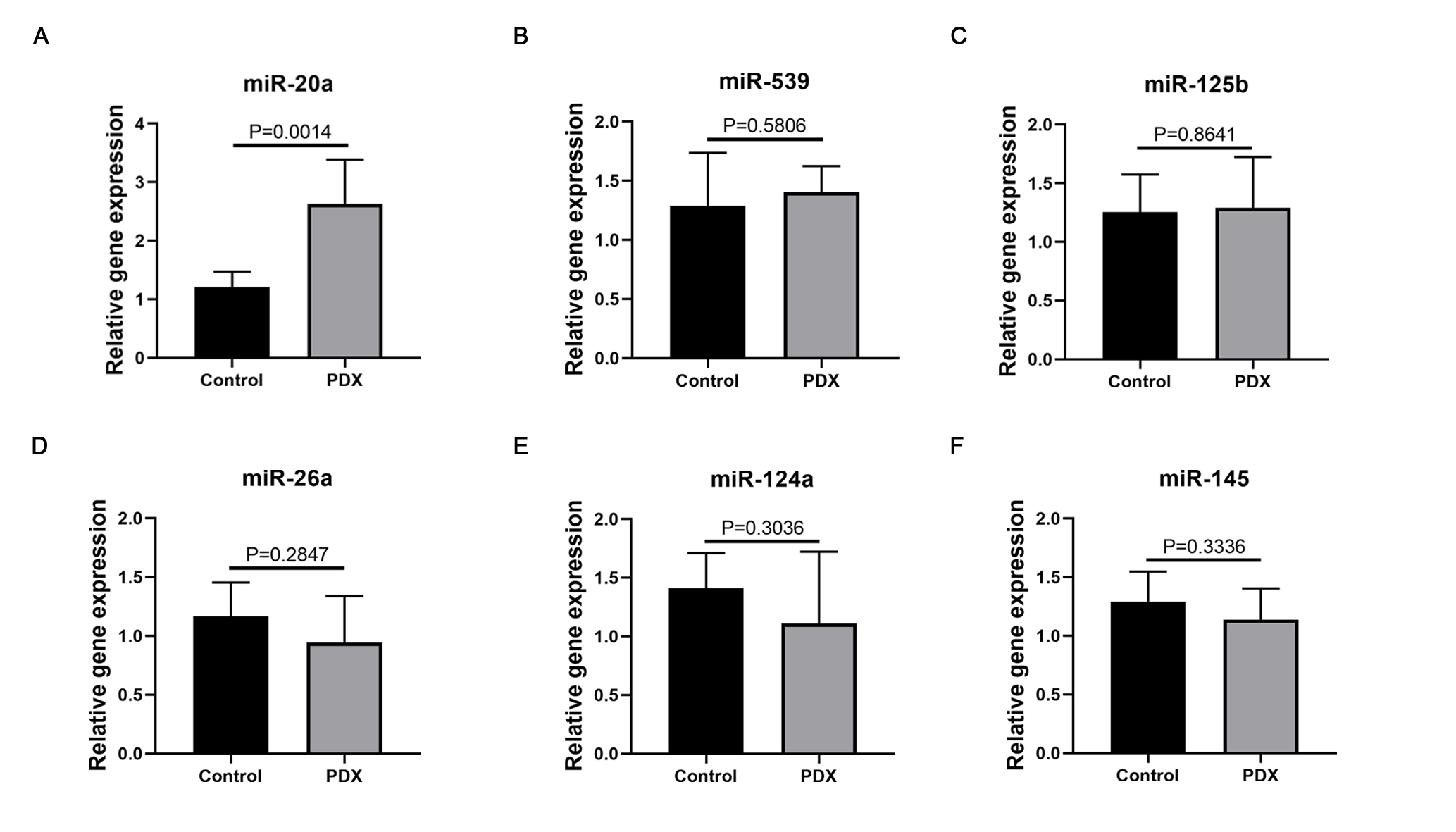

Supplement: Supplementary file 2 — Figure S2. (A-F) The expression of miR-20a, miR-539, miR-125b, miR-26a, miR-124a and miR-145 in BMDMs treated with PDX was verified by RT-qPCR. [file 41419_2021_3562_MOESM2_ESM.tif]
