# Supplementary material for: Protectin DX restores Treg/Th17 cell balance in rheumatoid arthritis by inhibiting NLRP3 inflammasome via miR-20a
Source: Cell Death Dis. 2021 Mar 15;12(3):280. doi: 10.1038/s41419-021-03562-6 (PMC7961047; doi:10.1038/s41419-021-03562-6)
Supplement: Supplementary file 3 — Table S1 Characteristics of patients with active RA, inactive RA and healthy controls. [file 41419_2021_3562_MOESM3_ESM.docx]

Table S1 Characteristics of patients with active RA, inactive RA and healthy controls

| Variable | Control  (n = 34) | Active RA  (n = 34) | Inactive RA  (n = 34) |
| --- | --- | --- | --- |
| Mean age (years) | 52.8 (15.45) | 60.3 (13.72) | 56.2 (15.87) |
| Female sex (%) | 26 (76.5) | 25 (73.8) | 26 (76.5) |
| Mean duration of rheumatoid arthritis (years) | NA | 6.6 (2.460) | 5.3 (2.738) |
| DAS 28 Mean score | NA | 3.2 (2.641) | 3.0 (2.188) |
| DAS 28 Score>5.1 (%) | NA | 12 (35.3) | 11 (32.3) |
| Mean C-reactive protein level (mg/L) | NA | 52.9 (19.08) | 12.6 (12.02) |
| Erythrocyte sedimentation rate (mm/h) | NA | 86.3 (12.51) | 44.8 (19.95) |
| Positive for rheumatoid factor (%) | NA | 25 (73.8) | 26 (76.5) |
| Positive for anti-CCP antibodies (%) | NA | 28 (82.4) | 28 (82.4) |

Data were presented with means (SD) or number (%).

RA = patients with rheumatoid arthritis; NA = not available
