# Supplementary material for: Protectin DX restores Treg/Th17 cell balance in rheumatoid arthritis by inhibiting NLRP3 inflammasome via miR-20a
Source: Cell Death Dis. 2021 Mar 15;12(3):280. doi: 10.1038/s41419-021-03562-6 (PMC7961047; doi:10.1038/s41419-021-03562-6)
Supplement: Supplementary file 4 — Table S2. Sequence(5’-3’) of primers for RT-qPCR and miRNA microarray. [file 41419_2021_3562_MOESM4_ESM.docx]

Table S2. Sequence(5’-3’) of primers for RT-qPCR and miRNA microarray

| NLRP3 | F | AGATTACCCGCCCGAGAAAG |
| --- | --- | --- |
|  | R | TCCCAGCAAACCCATCCACT |
| β-actin | F | CCTTCCTTCTTGGGTATGGA |
|  | R | ACGGATATCAACGTCACACT |
| has-miR-26a | F | TTCAAGTAATCCAGGATAGGCT |
|  | R | TGGTGTCGTGGAGTCG |
| has-miR-20a | F | TAAAGTGCTTATAGTGCAGGTAG |
|  | R | TGGTGTCGTGGAGTCG |
| has-miR-539 | F | GAAGAGGCTAACGTGAGGTTG |
|  | R | CACCATGACCAAGCCACGTAG |
| has-miR-145 | F | GTCCAGTTTTCCCAGGAATCCCT |
|  | R | CAGGTCAAAAGGGTCCTTAGGGA |
| has-miR-124a | F | GTGCAGGGGTCCGAGGT |
|  | R | AAGGCACGCGGTGAATGC |
| hsa-miR-125b | F | GAAGAAATACCATACCACCTGTT |
|  | R | GTCACCTGATCCCATCTAACAAT |
| U6 | F | CTCGCTTCGGCAGCACATATACT |
|  | R | ACGCTTCACGAATTTGCGTGTC |
